# Comparing unilateral and bilateral upper limb training: The ULTRA-stroke program design

**DOI:** 10.1186/1471-2377-9-57

**Published:** 2009-11-06

**Authors:** A (Lex) EQ van Delden, C (Lieke) E Peper, Jaap Harlaar, Andreas Daffertshofer, Nienke I Zijp, Kirsten Nienhuys, Peter Koppe, Gert Kwakkel, Peter J Beek

**Affiliations:** 1 Research Institute MOVE, Faculty of Human Movement Sciences, VU University Amsterdam, Van der Boechorststraat 9, 1081 BT Amsterdam, The Netherlands; 2 Research Institute MOVE, Department of Rehabilitation Medicine, VU University Medical Center, De Boelelaan 1117, 1081 HV Amsterdam, The Netherlands; 3 Rehabilitation Centre Amsterdam, Overtoom 283, 1054 HW Amsterdam, The Netherlands

## Abstract

**Background:**

About 80% of all stroke survivors have an upper limb paresis immediately after stroke, only about a third of whom (30 to 40%) regain some dexterity within six months following conventional treatment programs. Of late, however, two recently developed interventions - constraint-induced movement therapy (CIMT) and bilateral arm training with rhythmic auditory cueing (BATRAC) - have shown promising results in the treatment of upper limb paresis in chronic stroke patients. The ULTRA-stroke (acronym for Upper Limb TRaining After stroke) program was conceived to assess the effectiveness of these interventions in subacute stroke patients and to examine how the observed changes in sensori-motor functioning relate to changes in stroke recovery mechanisms associated with peripheral stiffness, interlimb interactions, and cortical inter- and intrahemispheric networks. The present paper describes the design of this single-blinded randomized clinical trial (RCT), which has recently started and will take several years to complete.

**Methods/Design:**

Sixty patients with a first ever stroke will be recruited. Patients will be stratified in terms of their remaining motor ability at the distal part of the arm (i.e., wrist and finger movements) and randomized over three intervention groups receiving modified CIMT, modified BATRAC, or an equally intensive (i.e., dose-matched) conventional treatment program for 6 weeks. Primary outcome variable is the score on the Action Research Arm test (ARAT), which will be assessed before, directly after, and 6 weeks after the intervention. During those test sessions all patients will also undergo measurements aimed at investigating the associated recovery mechanisms using haptic robots and magneto-encephalography (MEG).

**Discussion:**

ULTRA-stroke is a 3-year translational research program which aims (1) to assess the relative effectiveness of the three interventions, on a group level but also as a function of patient characteristics, and (2) to delineate the functional and neurophysiological changes that are induced by those interventions.

The outcome on the ARAT together with information about changes in the associated mechanisms will provide a better understanding of how specific therapies influence neurobiological changes, and which post-stroke conditions lend themselves to specific treatments.

**Trial Registration:**

The ULTRA-stroke program is registered at the Netherlands Trial Register (NTR, http://www.trialregister.nl, number NTR1665).

## Background

In the Netherlands, each year more than 32,000 patients sustain a stroke [1,2], and the incidence is expected to have increased by 30-45% by 2015 [3]. About 80% of stroke survivors suffer from an upper limb paresis immediately after stroke [4], hampering movement of the paretic arm and bimanual coordination [5].

Spontaneous recovery after stroke is limited, and knowledge about which mechanisms lead to spontaneous recovery is incomplete [6]. Restitution of non-infarcted penumbral tissue (i.e., reestablishment of metabolism in the tissues surrounding the infarcted area) [7] and resolution of diaschisis (i.e., relief of suppression of anatomically related brain areas) [8], together with recovery of neurotransmission in spared tissue near and remote from the infarct [7,8], are held mainly responsible for the nonlinear recovery pattern observed in the first weeks post-stroke [6].

In addition to these early post-stroke developments, functional recovery of the upper extremity is promoted by plastic changes in the functioning of the brain, which, in general, also occur in learning [9]. These experience-induced changes are brought about by a combination of neural repair and neuro-anatomic reorganization, and include greater excitability and recruitment of the neurons in both hemispheres, sprouting of dendrites, and strengthening of synaptic connections [7,10-16].

Although the aforementioned processes may suggest an optimistic view on post-stroke recovery, only one third of all stroke patients regain some dexterity within six months using conventional treatment programs [7]. This means that 60-70% of all stroke survivors will continue to experience major functional limitations of the upper extremity [17], which are associated with diminished health-related quality of life after stroke [7,18].

In light of this grim prospect, it is encouraging that recent studies, capitalizing on the concept of experience-induced neuroplasticity, have produced promising results using specific interventions aimed at arm-function improvement. One such intervention is bilateral arm training with rhythmic auditory cueing (BATRAC), which has been shown to have beneficial effects on the paretic arm in chronic stroke patients [19], possibly as a result of changes in contralesional cortical networks [20]. This suggests that motor function in the impaired paretic arm may be regained by exploiting interhemispheric interactions [21]. In particular, based on the principle of interhemispheric recruitment from the non-affected hemisphere (i.e., exercise-induced neuroplasticity by means of "neural cross-talk"), BATRAC may serve as an effective therapy for patients in whom the corticospinal tract (CST) system is seriously affected [22] - a group of patients for which effective therapies are urgently lacking and prospects of arm function recovery are particularly poor [8,17]. Furthermore, a recent meta-analysis on upper limb robotics suggests that distally oriented repetitive bilateral arm training is more effective than a more proximally oriented approach [23]. In addition, longitudinal studies with repeated measurements in time suggest that an early return of wrist and finger extension is a pre-requisite for regaining some dexterity [24-26]. These findings support the hypothesis that the effectiveness of BATRAC may be enhanced by performing repetitive flexion and extension movements of wrist and fingers, rather than rhythmic movements of more proximal parts of the arm.

In contrast, various controlled trials have suggested that intensive unilateral training by constraining movements of the non-paretic arm (constrained-induced movement therapy, CIMT) is an effective method for improving upper limb function in chronic stroke patients [18,27-29]. This suggests that training may also induce beneficial changes in the affected rather than the non-affected hemisphere and raises the question whether the improved functionality of the paretic arm with BATRAC indeed results from exploiting interhemispheric interactions, or merely from training with the affected arm [20].

The ULTRA-stroke program entails a randomized clinical trial (RCT) in which the merits of both BATRAC and CIMT are compared with each other and those of an equally intensive (i.e., dose-matched) conventional treatment program. To this end, participants will be divided over three intervention groups and the effects of the interventions will be assessed prior to training (t0), after 6 weeks of training (t1), and 6 weeks after training (t2). The primary aim of the ULTRA-stroke program is to assess the relative effectiveness of the three interventions on a group level and as a function of patient characteristics. In addition, the program aims for delineating the functional and neurophysiological changes that are induced by those interventions. This led to the following research questions:

▪ Which of the three interventions - modified BATRAC, modified CIMT, or a dose-matched conventional treatment (DMCT) - is more effective in terms of recovery of (unimanual and bimanual) hand and arm function in subacute stroke patients?

▪ How are the observed changes in functionality related to changes in peripheral stiffness, interlimb interactions, and cortical inter- and intrahemispheric networks?

The effectiveness will be assessed by a range of functional outcome measures pertaining to motor ability of the paretic arm, activities of daily living (ADLs), bimanual coordination, and peripheral motor functioning. Besides further elucidating the merits of bilateral versus unilateral upper limb training in general, the study will generate specific insights into the effectiveness of distally oriented (modified) BATRAC, specifically aimed at improving wrist and finger extension [24-26], and into the effectiveness of (modified) CIMT as applied in a thus far hardly studied stage after stroke [30].

In light of contrasting results and divergent perspectives regarding underlying mechanisms of current interventions [31], their potential dependence on the neurological characteristics of stroke survivors will also be a topic of investigation in the ULTRA-stroke program. It has been proposed that the effectiveness of CIMT is dependent on CST integrity [32-34], which is essential for motor control of the distal part of the upper limb. On the other hand, BATRAC may be expected to be less dependent on the integrity of the CST, as it appears to induce reorganizations in cortical inter- and intrahemispheric networks [21,22]. To cope with this issue, participants will be categorized in terms of their motor ability of the distal part of the arm [8].

In short, we hypothesize that both modified CIMT and modified BATRAC significantly improve upper limb function when compared to DMCT. Modified CIMT is expected to have a larger impact on those subjects who already showed some dexterity at recruitment than on subjects that were more restricted in this regard, given the proposed importance of CST integrity for motor control of the distal part of the upper limb [25]. Modified BATRAC, on the other hand, is expected to be also effective for the latter group of subjects, thanks to influences stemming from and reorganizations in the contralesional hemisphere (see also [35-37]). The effects of modified BATRAC and modified CIMT are both expected to sustain during the follow-up period of 6 weeks. To uncover the mechanisms associated with intervention-induced functional improvement, three kinds of analysis will be included.

First, endpoint mechanical behavior of the paretic wrist, under both passive and active conditions, will be assessed to determine both paresis and stiffness, the latter described in terms of intrinsic visco-elasticity and reflexive feedback properties [38]. In *active *posture tasks the negative signs of post-stroke limb dysfunction prevail (viz., paresis and poor adaptation of reflexes; [39]). Under *passive *conditions, however, enhanced joint stiffness and hyper-excitability of the reflex loop (viz. enhanced tendency for synchronous and self-sustained firing of the motor neuron pool) are evident [40,41]. Because the spinal reflex loop is under control of higher brain areas, loss of CST integrity and persistent central nervous system reorganization is anticipated to be related to high joint stiffness, absence of reflex modulation, and signs of hyper-excitability of the reflex loop. Assuming that increased joint stiffness is specifically associated with loss of CST integrity, modified BATRAC is expected to be more effective in reducing these effects than modified CIMT for participants with minimal hand/finger control.

Second, bimanual coordination will be examined in all detail. Bimanual coordination is characterized by interlimb interactions [42,43] that result in stabilization of specific bimanual coordination patterns [5,44-49]. The success of bilateral training protocols (such as BATRAC) has been ascribed to the presence of such interlimb interactions [21], suggesting that influences from the contralesional hemisphere are beneficial for performance of the paretic limb. Bilateral training may also induce adaptations in these interactions, potentially strengthening its advantageous influence on paretic arm performance as well as improving bimanual performance. Therefore, modified BATRAC is expected to induce more improvement in these interactions than both modified CIMT and DMCT.

Third, treatment-induced neuronal reorganization will be identified using magneto-encephalographic recordings (MEG). Given its high temporal resolution, MEG is a very suitable non-invasive tool for studying patterns of correlated neuro-electrical activity within and across hemispheres. MEG recordings of unimanual and bimanual tasks will be conducted prior to and after interventions to investigate treatment induced changes in these patterns.

Functional MRI studies and TMS studies already indicated that, during paretic arm movement, CIMT results in increased metabolic activation in the primary sensorimotor cortex of the affected hemisphere [50-62], whereas BATRAC results in increased metabolic activation in the contralesional cerebrum and ipsilesional cerebellum [20,21].

Modified CIMT is hence expected to result primarily in changes in ipsilesional hemisphere functioning, i.e., greater activity in the primary sensorimotor cortex of the affected hemisphere and increased phase synchronization between regions surrounding the lesion (we note that assessing the latter requires the high temporal resolution of encephalographic recordings), which may be related to restitution of its former functionality. In contrast, modified BATRAC is expected to primarily induce adaptations in the contralesional hemisphere, enhanced activity in the (ipsilesional) cerebellum (possibly reflecting enhanced timing abilities), and a considerably greater increase in the degree of phase synchronization between the lesioned hemisphere and the contralesional hemisphere than will occur as a result of either modified CIMT or DMCT. This finding would indicate compensatory cortical reorganizations in which the coupling to the nonaffected hemisphere acquires a special role in the motor control of the paretic arm.

## Methods

The ULTRA-stroke program has been approved by the Medical Ethical Reviewing Committee of the VU University Medical Centre (protocol number 2008/296, Dutch Central Committee on Research Involving Human Subjects, CCMO, protocol number NL20456.029.08).

### Recruitment

Sixty patients, admitted to the Rehabilitation Centre Amsterdam (RCA), who meet the criteria within 6 months after stroke onset will be recruited. Both in- and outpatients will be enrolled.

The inclusion criteria are: a first-ever ischemic or hemorrhagic stroke in one of the hemispheres, as verified by CT and/or MRI scan; an upper limb deficit, however with minimal control of the paretic wrist and fingers (i.e., able to execute at least 10° of active wrist extension, at least 10° of thumb abduction/extension, and at least 10° extension in at least 2 additional digits); a score on the Action Research Arm Test (ARAT) of less than 53 points; between 18 and 80 years of age; written or oral informed consent; sufficient motivation to participate.

The exclusion criteria are: upper extremity orthopaedic limitations; not being able to communicate (i.e., < 4 points on the Utrecht Communication Observation, UCO [63]); a Mini Mental State Examination (MMSE) score of < 24 points [64]. Patients with a pacemaker or metallic implant will be recruited. However, they will not be subjected to MEG-recordings given the interference with the magnetic signal. There will be no restrictions with respect to gender, ethnicity, or socio-economic status.

### Design

The intake procedure will take place the week following informed consent. After the intake procedure, which will include the first assessment of outcome variables, patients will be stratified in those with some dexterity and those with minimal control of paretic wrist and finger extension. Patients with some dexterity are able to execute > 10° finger extension of each metacarpophalangeal and interphalangeal joint of all digits and > 20° wrist extension. Patients with minimal control are those who meet the criteria of inclusion (i.e., able to execute at least 10° of active wrist extension, at least 10° of thumb abduction/extension, and at least 10° extension in at least 2 additional digits), but not (yet) the criteria of some dexterity. After stratification, patients will be randomized in permuted blocks and allocated to one of the three intervention groups (i.e., modified BATRAC, modified CIMT, or DMCT). Concealed allocation is effectuated with an online, computerized randomization procedure according to the minimization method [65]. Other therapists and social workers will provide regular care depending on patient needs.

**Figure 1:**
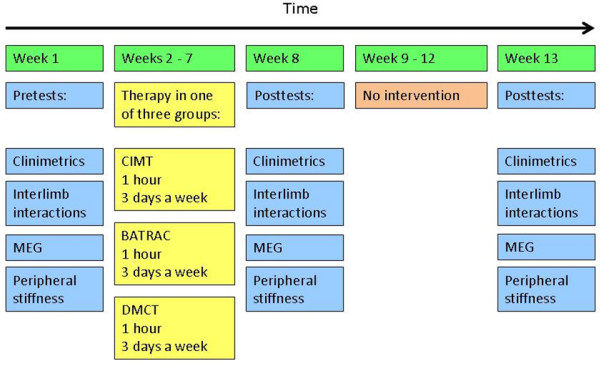
**Timepath**. Participant flow through the trial.

After the first assessment and randomization, an intervention period will take place for 6 consecutive weeks. The effects of the interventions are examined using a pretest-posttest design. The pretests (t0) are performed in the week prior to intervention and posttests (t1) are performed in the week after intervention. The degrees to which changes are sustained are examined using retention tests (t2), 6 weeks after completion of the intervention. Figure 1 shows the time schedule for effectuating the entire protocol. (Figure 1.)

### Interventions

The interventions will be applied by physiotherapists and/or occupational therapists working at the RCA. If possible, interventions will be applied in groups with no more than 3 patients per group in a treatment session.

### Modified BATRAC

The modified BATRAC group receives 60-minute sessions, 3 days a week for 6 consecutive weeks. Treatment will be applied in 3-minute movement periods interspersed with 5-minute rest periods (i.e., effectively 21 minutes of active movements). During the rest periods and before the first exercise, patients will receive visual and oral feedback on the previous exercise (when applicable) and instructions for the following exercise. The movements during the exercises are paced by an auditory metronome. The tempo of the auditory cues depends on the severity of the upper limb deficit and is selected individually. Over the course of training the tempo is adjusted in response to improvement in task performance.

#### Apparatus

**Figure 2:**
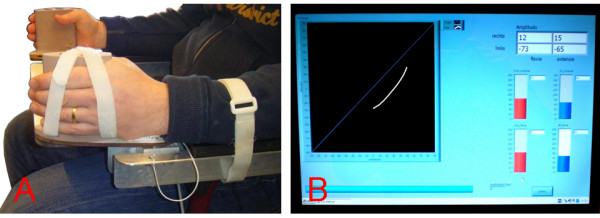
**Modified BATRAC exercise apparatus**. A: the modified BATRAC exercise apparatus. The apparatus allows movements in the horizontal plane only. The rotation axis of the wrist is aligned with that of the manipulandum B: a feedback screenshot. On the left, left-hand position by right-hand position (white) and ideal line of movement are depicted. On the top-right the amplitudes for flexion and extension for both hands separately are given. On the bottom right the relative phase and its variability for flexion and extension are presented.

During therapy, custom-made modified BATRAC-apparatuses (developed at the Faculty of Human Movement Sciences of VU University Amsterdam) will be used (see Figure 2A). The apparatus is mounted on a chair with arm rests. At the distal end of each arm rest, a manipulandum with a handgrip is fitted which allows motion in the horizontal plane. In front of the patient, between the arm rests, a removable table top is placed. The patient is seated on the prepared chair, with the ankles in neutral dorsiflexion and knees and hips placed at 90°. The patient's hands are vertically fixated to the handgrips and the lower arms are fixated to the arm rests with Velcro straps. These fixations, together with an adjustable stop at the proximal end of the arm rests, allow flexion and extension movements of the wrist only. The distance of the handgrip on the manipulanda is adjustable to make sure the rotation axis of the wrist is aligned with that of the manipulandum.

Below the manipulanda, potentiometers (FCP40A, tolerance ± 0.1%, Sakae Tsushin Kogyo Co., Ltd., Nakahara-ku, Kawasaki-city, Japan) are attached that measure the movements (i.e., wrist rotation) during exercise. A computer connected to the potentiometers registers these movements and provides feedback. The computer is also used to start each exercise, i.e., exercise type (see below) and pacing frequency are set via the computer. The auditory cues (duration 50 ms, pitch 440 Hz) are generated by the computer and presented through headphones. (Figure 2.)

#### Goals

BATRAC has been motivated from research on the interlimb interactions governing bimanual coordination. Bimanual coordination is characterized by both spatial and temporal interactions [43,66]. The interactions become apparent when two limbs are instructed to move at unequal frequencies [49] or amplitudes [67] or by the fact that for isofrequency coordination (i.e., the limbs move at identical frequencies) only two coordination patterns can be performed stably without practise: in-phase (i.e., the limbs oscillate symmetrically) and antiphase (i.e., the limbs oscillate in an alternating fashion) [68].

The primary goal of modified BATRAC is to optimize the coordination between both hands. In the context of BATRAC, this means that temporal coupling between rhythmic hand movements (i.e., flexion and extension movements) in the two intrinsically stable coordination patterns (i.e., in-phase and antiphase) will be utilized. To achieve optimization of coordination, a large number of repetitive movements will be performed at a specified frequency during the exercises outlined below.

Although these exercises may seem unrelated to the functional goal of regaining voluntary control over hand function in daily situations, their motivation resides in the conjecture that hand function improves when the least-affected hemisphere facilitates controlling the movements of the paretic arm. Following this principle, optimization of coordination between both upper extremities is beneficial for regaining functional ability and functional use [19,20].

The second goal of modified BATRAC is to increase the range of motion of the wrist, with a strong emphasis on active wrist and finger extension. Loss of hand function is problematic because it is crucial to manual exploration and manipulation of the environment, and thus a major source of disability in stroke. The increase in force control associated with the generation of larger movement amplitudes, especially towards wrist and finger extension, will contribute to fine motor control, as this is required in manipulating and releasing grasped objects.

#### Exercises

**Figure 3:**
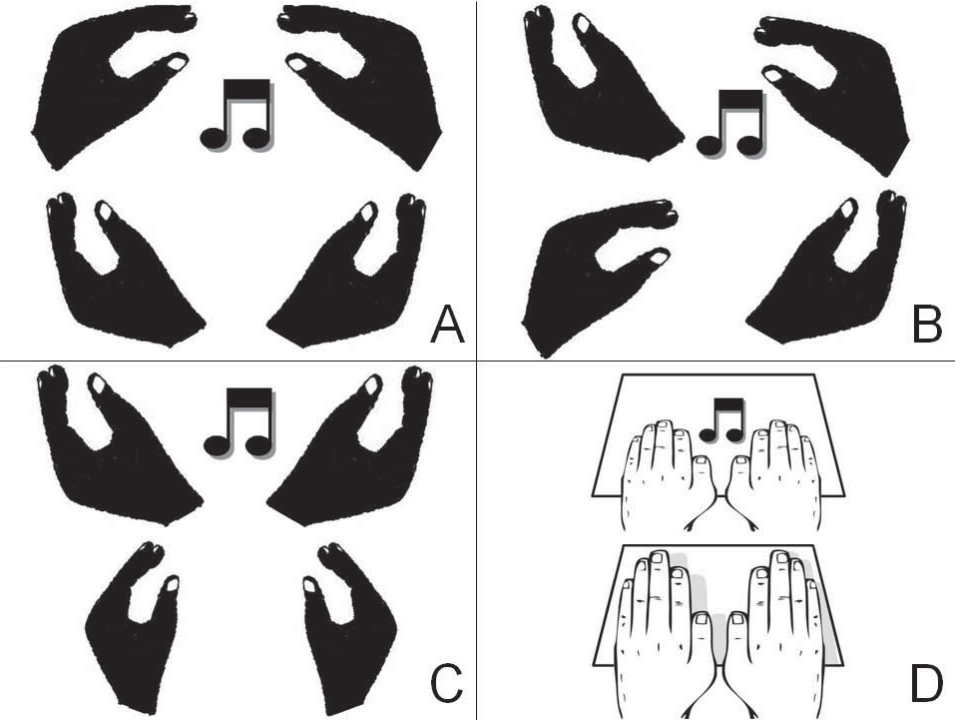
**Modified BATRAC exercises**. A: in-phase exercise with flexion of both hands on the cue. B: antiphase exercise with simultaneous right-handed flexion and left-handed extension on the cue for half of the antiphase exercises (vice versa for the other half). C: extension exercise with extension of hands on the cue. D: music exercise with tapping of both hands on the beat.

With the described goals in mind, therapists can choose from four different exercises (see Figure 3). First, in the *in-phase *exercise patients move both hands simultaneously towards flexion followed by a movement towards extension. These movements should follow each other smoothly and rhythmically. Maximal flexion should occur at the moment of the metronome cue. The pacing frequency is set, so that the patient can easily complete the required 3 minutes of exercise time.

Second, in the *antiphase *exercise patients move both hands simultaneously to the left followed by a movement to the right. These movements should follow each other smoothly and rhythmically. Maximal flexion of the right hand and maximal extension of the left hand should occur at the moment of the cue in half of the exercises, and vice versa in the other half. Again, the frequency is chosen such that the patient can comfortably complete the 3 minutes exercise time.

Third is the *extension *exercise. Patients move both hands simultaneously towards extension; maximal extension should occur at the moment of the cue. In this exercise extension activity is emphasized, such that active movements towards flexion are not required: after each cued maximal extension, patients can relax the wrist extensors and let the hands fall back to a neutral position. Again, the patient should comfortably complete the 3 minutes of exercise time with the chosen frequency.

Finally, in the music exercise the manipulanda are not used; instead, the patient's hands are placed (palms down) on the table top between the armrests. Through the headphones patients will hear a song in which the beat, with a specific and constant frequency, is emphasized by slightly louder pitches. There are several songs to choose from with pacing frequencies ranging from 0.8 to 1.4 Hz. Patients are instructed to raise their fingers and hand, while keeping the base of the hand resting on the table, and tap the hand on the table on the beat of the music. This can be done either in-phase or in antiphase.

As stated above, in each exercise the pacing frequency is set at a frequency that allows the patient to comfortably complete the 3 minutes exercise time. Because metronome beats at frequencies below 0.67 Hz (and above 4 Hz) are not perceived as rhythmic patterns [69,70], a minimum frequency of 0.8 Hz is used in the exercises. To enhance the training effect, however, the actually used pacing frequency will be the highest possible comfortable frequency for each individual patient, and may be increased over the intervention sessions. Therapists are advised to vary the exercises during each session to enhance therapy compliance. However, in-phase exercises are prioritized over antiphase exercises. In-phase bimanual movement patterns are most stable [45] and are therefore assumed to benefit most from interhemispheric interaction [71,72]. Patients with difficulties to keep track of the pacing frequency are encouraged to facilitate the rhythmic movements, for instance by counting and foot tapping. Tempo, types of exercise, and performance will be recorded in a patient-log to keep track of the course during the 6-week intervention period. Patients are also advised to practice rhythmic bimanual exercises, like clapping to music beats, in their own time. (Figure 3.)

#### Feedback

The computer connected to the modified BATRAC-apparatus provides feedback about the patient's performance. During the exercise, a diagonal line in a left-hand position by right-hand position plot marks the ideal line of coordination. The actual movement is presented by a moving dot with a 4 cm trailing tail (see Figure 2B). In early stages of the intervention, this form of feedback is not presented to the patient during the exercise to avoid attentional interference. In later sessions, when the patient is accustomed to the exercise, this form of visual feedback will be used to improve bilateral coordination.

After each exercise, the maximal amplitudes towards flexion and extension of both hands, and the relative phase between both hands (Φ, [73]) and its variability are presented in numbers and diagrams (see Figure 2B). These are recorded in a patient-log and will be used to motivate the patient to improve performance in following exercises.

### Modified CIMT

Like the modified BATRAC group, the modified CIMT group receives 60-minute therapy sessions, 3 days a week for 6 consecutive weeks. Modified CIMT involves functionally oriented task practice of the paretic arm and hand, while the less-impaired hand is restrained for 6 hours each weekday, hence also during therapy.

#### Goals

Modified CIMT is aimed at progressively improving motor task performance of the paretic arm and hand. A premise for this improvement is the prevention or turnabout of learned non-use [29,74-78]. A specific goal is to increase control over active extension of wrist and fingers, as this ability is important for fine motor control in functional tasks [24,25].

#### Exercises/matrix

Modified CIMT techniques include repetitive functional task practices and shaping of the desired improvements of movement using the technique of successive approximation (i.e., breaking complex movements into steps) [74,76]. During exercise the patient receives continuous verbal feedback and stimulation and, if necessary, hands-on facilitation of movements.

**Figure 4:**
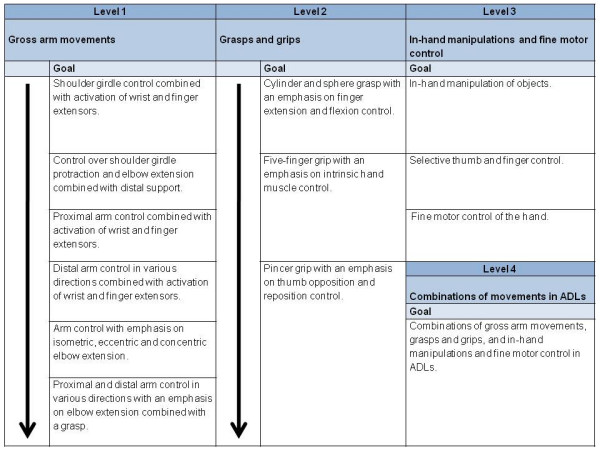
**Quasi-hierarchical modified CIMT Matrix**. The matrix functions as a tool for therapists. Exercise difficulty increases from top to bottom and from left to right. Patients should train on their highest possible level.

The exercises follow a quasi-hierarchical bottom-up approach from more easy applied gross motor functions to more complex in-hand manipulations. Figure 4 represents the matrix that serves as a guideline for building-up the modified CIMT exercise program. In this matrix the trained functional movements are described at 4 functional levels: gross arm movements, grasps and grips, in-hand manipulations and fine motor control, and combinations of movements in ADLs. The matrix follows a hierarchical pattern. As can be seen in the matrix, there is no hierarchy within the third level. Preferably, patients should train on the highest possible level and engage exercises that demand the most of their possibilities. Task difficulty within each exercise is varied by adjusting spatial or temporal demands. The content and duration of each session as well as the shaping exercises are recorded in a patient-log reflecting the progress in reaching treatment goals. (Figure 4.)

#### Mitt

To encourage the use of the most affected arm and hand in ADLs, a padded safety mitt (Sammons Preston # 6727; Sammons Preston, Inc., Bolingbrook, IL, USA) is applied to immobilize the less affected hand for at least 6 waking hours each weekday. A therapist, nurse, or family member should help putting on and taking off the mitt. The mitt should be taken off during bathroom activities. Patients that go walking by themselves are only allowed to wear the mitt if they score more than 3 points on the Functional Ambulation Categories (FAC). The mitt prevents contracture with firm polyester filling and allows (preventive) elbow extension, leaving sufficient movement when needed, for example in case the patient threatens to fall.

### DMCT

DMCT is an exercise therapy based on existing guidelines for upper extremity treatment after stroke as presented by the Dutch Society of Occupational Therapy [79] and the Royal Dutch Society of Physical Therapy [80]. This exercise therapy is typically provided by therapists at the RCA. However, specific elements of (modified) BATRAC (e.g., rhythmic cues) and (modified) CIMT (e.g., use of mitt) will not be used.

Therapy will be provided 60 minutes per treatment session, 3 days a week for 6 consecutive weeks, and will not contain specific elements of the other two therapies. The content and duration of the sessions are recorded in a patient's log.

### Power analysis

The number of patients is based on a statistical power of 80% (preventing Type II error) with an alpha of 5% (preventing Type I error) for detecting a meaningful difference of 6 points on the ARAT as the primary measurement of outcome and expecting 15% drop-out. The statistical power for detecting 10% or 6 points difference between groups is based on the following power calculation:

(Z_1-α/2 _+ Z_1-β_)^2 ^= 10.43 (assuming an alpha of 0.017, corrected for multiple testing with 3 groups and a Beta of 0.80)

σ2 (expected variance of sample on ARAT) = 26

Δ2 (assumed difference in favour ARAT) = 36

r (expected ratio between intervention groups) = 1

Expected numbers needed per intervention group (N_per group_) = 15 (excluding expected drop out of less than 25%).

### Outcome variables

The primary outcome variable will be the score on the Action Research Arm test (ARAT). Secondary outcome variables are the scores on the Motricity Index (MI) of the arm, Fugl-Meyer for the arm (FM-arm), Nine Hole Peg test (NHPT), Erasmus modification of the Nottingham Sensory Assessment (EmNSA), Motor Activity Log (MAL), and Stroke Impact Scale (SIS version 3.0). All assessments will be applied to all patients at t0, t1, and t2.

Non-parametric statistics for independent samples will be used [81]. A Kruskal-Wallis test will be used to examine whether improvements before and after intervention (from t0 to t1) were significantly different between the three intervention groups (i.e., BATRAC, CIMT, and DMCT). When a significant difference is found between the three groups, a *post-hoc *Mann-Whitney U test will be applied to reveal which groups (CIMT, BATRAC, or DMCT) differ significantly from each other. The same procedure will be used for observed changes after the 6 weeks retention period, i.e., from t1 to t2.

The ARAT is a valid, reliable, and responsive performance test [82] of the ability to perform gross movements and to grasp, move, and release objects differing in size, weight, and shape [83]. The minimal clinically important difference is set at about 10% of the scale's range, i.e., 6 points [84]; improvement by > 10 points is defined as return of dexterity [17].

The MI will be used to measure strength in upper extremities [85]. Higher scores represent greater strength in the upper limb. This instrument provides a reliable and valid assessment of the presence of paresis in stroke patients by testing 6 functions rated for each limb [64,85].

The FM arm score is a reliable and valid motor performance test consisting of 33 tasks performed by the affected upper limb [86,87]. The FM-arm test evaluates the ability to make movements outside the synergistic pattern. Performance on each task is rated as 0, 1, or 2, with higher rates representing better performance. The FM-arm measure will be used as the sum of 33 ratings (possible range 0 to 66 points).

The NHPT is a reliable and valid test that measures manual dexterity [88,89]. It measures the speed with which a patient grasps and inserts (and removes) 9 pegs into a grid of vertical holes. The test will be discontinued after 50 s if the patient is still unable to insert any pegs. The NHPT measure for each hand is calculated by the number of pegs placed per second. The affected as well as the unaffected hand will be measured. Reliability and validity have been assessed and norms are available [88,89].

The EmNSA is a 3 point ordinal scale that measures sharp-blunt discrimination, two-point discrimination, and limb proprioception. The EmNSA will be restricted to the paretic upper limb (i.e., fingers, hand, and forearm). With the exception of the two-point discrimination item, intra- and interrater reliability of tactile sensations, sharp-blunt discrimination and proprioception items are good to excellent (Kappa: 0.58 to 1.00, [90]).

A translated and adapted version of the MAL will be used [91], which contains the 14 original activities, 11 additional activities, and 1 optional activity chosen by the patient. Reliability and validity of the MAL has been demonstrated in a number of studies [91]. The MAL will be administered to each applicant and, if available, their caregivers. It will be used to independently rate how well (5-point Quality of Movement [QOM] scale) and how much (5-point Amount of Use [AOU] scale) the paretic arm was used spontaneously to accomplish 26 activities of daily living outside the laboratory [84,92].

The arm-hand domain of the SIS (version 3.0) will be used to evaluate patients' perceived outcome for the paretic upper limb. Version 3.0 of the SIS is a full-spectrum health status interview that measures changes in 8 impairments, function and quality-of-life subdomains following stroke and will be used as a secondary outcome measure [93]. Each domain will be analyzed separately. The upper limb part of the SIS includes 5 questions about patients' perceived competency to keep their balance, to transfer, to walk in the house and negotiate stairs, to get in and out of a car, and to move about in their own community. Each item is scored from "not difficult at all" to "cannot do at all" on a 5 point rating scale. A difference of 5 points (10%) on the "hand function" domain of the SIS is perceived as clinically relevant [28]. The SIS has shown excellent clinimetric properties in terms of concurrent and construct validity, test-retest reliability, and responsiveness [94].

### Associated mechanisms

To delineate the functional and neurophysiological changes that are associated with the effects of BATRAC and CIMT, three additional tests will be administered before and after intervention, and after the retention period.

### Peripheral stiffness

Endpoint mechanical behavior of the paretic wrist, resulting from a mix of visco-elastic (intrinsic) and proprioceptive reflex (reflexive) properties, will be assessed under both passive and active conditions using a haptic robot ('Wristalyzer', Moog FCS Inc., Nieuw-Vennep, The Netherlands). This powerful, force-controlled manipulator applies force- and position perturbations to the paretic wrist, while the interaction with the patient, in terms of forces and joint angles, is measured. A haptic controller, which overrules the dynamics of the manipulator with imposed virtual dynamics, is used to apply perturbations. The result is that, to the patient, the manipulator behaves like a mass-spring-damper system. After assessment of the range of motion (ROM) and maximal force, both towards flexion and extension, a number of perturbation tests under both passive and active conditions will be applied. Using system identification in the frequency domain [39,95,96], the intrinsic vs. reflexive stiffness is calculated from the time series of net moment and wrist position. Surface EMG (16-channel Biotel 99 EMG amplifier, Glonner Electronic GmbH, Munich, Germany) is measured for additional validation. From the time records of position and force, frequency response functions (FRFs) will be estimated. FRFs are transfer functions that express the structural response to an applied force in the frequency domain. Given a model structure and an appropriate estimate of the intrinsic component, an estimate of the reflex gains for length and velocity can be obtained from the FRFs [39,95,96]. For both intrinsic and reflexive properties, a repeated measures AN(C)OVA will be performed to detect differences over time and between groups (intervention/stratification).

### Interlimb interactions

Ridderikhoff et al. [71] devised an experimental methodology to dissociate between the contributions of three sources of interlimb interaction: integration of feed-forward control signals to both hands; error correction of the phasing between the hands, based on afferent signals; and (unintended) phase entrainment by contralateral afferent signals, probably resulting from spinal reflexes. It is based on systematic comparisons between four coordination tasks involving bimanual performance (in- and antiphase coordination) and unimanual performances with and without comparable motor-driven movements of the contralateral hand. The four tasks are the following: (a) unimanual rhythmic coordination with an auditory pacing signal (UN); (b) idem, while the contralateral hand is moved passively with a phase shift of 30° with respect to the required movements of the active hand (UNm); (c) kinesthetic tracking (KT): unimanual active movements are to be coordinated (in- or antiphase) with the passive rhythmic movements of the contralateral hand; and (d) active auditorily-paced bimanual coordination (in- or antiphase; AB). The tasks are performed in an experimental set-up in which passive movements can be imposed using a servo-motor (Parvex RS440GR1031, SSD Parvex SAS, Dijon Cedex, France) and a precision gearbox (alpha TP010S-MF1-7-0C0, backlash ± 0.02°, Wittenstein, Inc., Bartlett, IL, USA), and the active movements are measured using potentiometers (FCP40A, tolerance ± 0.1%, Sakae Tsushin Kogyo Co., Ltd., Nakahara-ku, Kawasaki-city, Japan). Depending on the task conditions, the motor-driven, passive movements are based on either the movements of the to-be-moved hand as recorded during condition (d), which is therefore the first task to be performed, or a predefined sinusoidal pattern (with added random noise). Given the inherent functional asymmetry in the subject population of interest, tasks (a)-(c) are performed with both the paretic and the non-paretic hand as active hand (order counterbalanced over subjects). The Analysis focuses on the relative phase between both hands in bimanual tasks (i.e., AB and KT) and between the active hand and the metronome in the unimanual tasks (i.e., UN and UNm). Mean relative phase and circular standard deviation will be used to dissociate between the contributions of three sources of interlimb interaction after each test separately (i.e., t0, t1, and t2; *t*-tests and repeated measures ANOVA), and to detect changes in these over the three tests (repeated measures AN(C)OVA). Additionally, correlations between the durations of simultaneously performed cycles (as an index of degree of coupling), and correlations between the error in the discrete relative phase and the duration of the following cycle of the non-affected hand (as an index of the effectiveness of error corrections), will be calculated for the tasks that involve two moving hands (i.e., AB, KT, UNm). These correlations will be transformed into normally distributed variables using the Fisher transform. A repeated measures AN(C)OVA will be used to examine the changes over time.

### Brain dynamics

To examine the changes in brain dynamics induced by the three intervention techniques, all patients perform simple unimanual and bimanual force production tasks with their hands, while whole-head MEG recordings will be made (CTF Systems Inc., Vancouver, Canada and Elekta Neuromag, Stockholm, Sweden). Task performance (i.e., onset and displacement of hand squeezes) will be monitored by pump bulbs (RXPUMPBULP, BIOPAC Systems, Inc., Goleta, CA, USA), tubing, and pressure transducers. The to be performed task will be as follows: 2 min of rest, 30 s of rhythmic squeezing on auditory cues, 30 s of rest, 30 s of rhythmic squeezing on auditory cues, 30 s of rest, 30 s of rhythmic squeezing on auditory cues, and finally 2 min of rest. Hence, the duration of a single task will be 6 min and 30 s. The frequency of auditory cues will be set at 1 Hz. The task will be performed once with the left hand only, once with the right hand only, and once bimanually. Surface EMG will be applied (using data acquisition channels built into the MEG system) to monitor task performance.

The MEG signals will be mapped from sensor space to source space using the synthetic aperture magnetronomy (SAM) minimum linear variance beamforming approach [97] to determine the brain regions that show the largest contrast when comparing activity pre- and post performance [98]. Changes in power in various frequency bands (theta, alpha, beta, and gamma) and cortico-cortical synchrony (using instantaneous Hilbert phase and the phase locking index; [99]) in these bands will be examined. For the associated analyses see Houweling et al. [98].

## Discussion

The ULTRA-stroke program is expected to have a strong impact on the treatment policies for stroke survivors. Given that about 80% of all stroke victims show upper limb paresis immediately following stroke [4], hampering unimanual and bimanual coordination [5], and the fact that only one third of all stroke patients will regain some dexterity within six months with conventional treatment programs [7], a sufficiently powerful RCT investigating the effectiveness of innovative therapies is relevant and urgently needed to objectively guide stroke rehabilitation.

Specifically, the combination of assessments of effectiveness and mechanisms associated with intervention-induced functional improvement will prove to be valuable for clinical practice. Therefore, next to the effectiveness of modified BATRAC and modified CIMT, the ULTRA-stroke program also intends to investigate how longitudinally changes in (a) upper limb neuromechanics (i.e., peripheral stiffness and interlimb interactions), and (b) neuroplasticity (i.e., changes in cortical inter- and intrahemispheric networks), are associated with recovery of motor impairment (i.e., synergism and strength) and upper limb function (i.e., dexterity). The outcome of the RCT, together with information about longitudinal changes in the underlying mechanisms of upper limb recovery, will provide clinicians a tool to make appropriate decisions in selecting evidence-based therapies for the paretic upper limb.

The ULTRA-stroke program is contingent upon the fact that the major part of post-stroke rehabilitation treatment takes place during the subacute phase. Since 2006, meta-analyses have shown that CIMT is an effective way to improve upper limb function in chronic stroke patients [18,27-29]. The same applies to BATRAC [19,20,100] (but see also [101]). However, high quality RCTs pertaining to the subacute phase post stroke have thus far been lacking in the literature [102]. Consequently, the ULTRA-stroke program is a unique and innovative project in stroke rehabilitation science.

As stated before, a better understanding of how specific therapies influence neurobiological changes, and more important, what post-stroke conditions lend themselves to specific treatments, will help clinicians to tune the treatment to the needs of the individual patient. The knowledge gained in the ULTRA-stroke program will further underpin the concepts of motor recovery and motor learning in stroke rehabilitation by addressing the key question: what changes in underlying mechanisms are associated with functional improvement after stroke?

Results of the ULTRA-stroke program are expected at the beginning of 2012.

## Competing interests

The authors declare that they have no competing interests.

## Authors' contributions

AEQ: is the executive investigator, prepared the first draft of the paper, and developed treatment protocols for CIMT and BATRAC. CEP: revised the manuscript critically, participated in the design and conceived of the study and its coordination, raised its funding, and contributes to the examination of interlimb interactions. JH: contributes to the examination of the peripheral stiffness. AD: contributes to the examination of brain dynamics. NIZ: participated in the development of treatment protocols, and coordinates the study and recruitment at the RCA. KN: participated in the development of treatment protocols, and coordinates the study and recruitment at the RCA. PK: coordinates the study and recruitment at the RCA. GK: revised the manuscript critically, participated in the design, conceived of the study and its coordination, and raised its funding. PJB: revised the manuscript critically, participated in the design, conceived of the study, raised its funding, and coordinates the study and its financial and technical support.

All authors read and approved of the manuscript.

## Pre-publication history

The pre-publication history for this paper can be accessed here:

http://www.biomedcentral.com/1471-2377/9/57/prepub
